# Effects of Application of Lactobacilli on Lipid Metabolism in Monogastric and Aquatic Animals: Benefits and Underlying Mechanism

**DOI:** 10.3390/ani16193111

**Published:** 2026-10-03

**Authors:** Bing Han, Christopher J. Andrews

**Affiliations:** 1 Key Laboratory of Feed Biotechnology of Ministry of Agriculture and Rural Affairs, Institute of Feed Research, Chinese Academy of Agricultural Sciences, No. 12 Zhong Guan Cun South Street, Haidian District, Beijing 100081, China; 2 Animal Science Group, School of Agriculture and Environment, Massey University, Private Bag 11-222, Palmerston North 4442, New Zealand

**Keywords:** lactobacilli, lipid metabolism, gut microbiota, animals, bile acids

## Abstract

Lipid metabolism disorder is a common physiological problem in monogastric livestock and aquatic animals which easily leads to excessive fat deposition, poor meat quality, and inhibited growth performance, bringing huge economic losses to the aquaculture and livestock industries. Lactobacilli, as core bacteria found in the gut of animals, play a crucial role in growth and health in monogastric animals, mainly including pigs, poultry, and pets, and even in aquatic animals. Nevertheless, existing relevant reviews mostly provide simple descriptive summaries of phenotypic effects, lacking systematic collation of strain-specific differences and multi-level regulatory mechanisms. Moreover, most mechanistic conclusions are predominantly inferred from rodent studies, while the applicability and specificity of these regulatory pathways in target livestock and aquatic animals remain unclear, forming an important research gap. In view of the above deficiencies, this review summarizes the beneficial effects of different lactobacilli on lipid metabolism in monogastric (including swine, poultry and pets) and aquatic animals. Furthermore, we elucidate the underlying mechanisms in different dimensions: short-chain fatty acid-mediated lipid signal transduction, regulation of liver–adipose axis-related genes (PPARs, SREBP-1c, FAS, etc.), and improvement in intestinal barrier function to inhibit lipid absorption. Also, considering the current limitations that existing mechanistic evidence in livestock and aquatic species is insufficient, this review highlights the bottlenecks of current research and proposes future research priorities, including species-specific mechanism verification, strain functional screening and precise application exploration. This study deepens the comprehensive understanding of lactobacillus-mediated lipid metabolism regulation and provides a reliable theoretical reference for the targeted application of lactobacilli in improving lipid metabolic disorders in monogastric animals and aquaculture animals.

## Simple Summary

Lipid metabolism disorders trigger excessive fat deposition and heavy economic losses in monogastric livestock and aquatic animals. While gut-derived lactobacilli can regulate lipid homeostasis, existing reviews lack systematic strain-specific analysis, and most mechanistic data are from rodent models. This review summarizes the lipid-modulating effects and underlying pathways of lactobacilli. Current research limitations and future priorities for their precise application in animal production are also highlighted.

## 1. Introduction

For decades, lactobacilli have been known to be a core group of beneficial bacteria found in the gastrointestinal tracts of animals. It is widely reported that lactobacilli could play important roles, such as modulating gut health [1,2], enhancing immunity [3], and maintaining microbiota balance in animals [4] including swine, poultry, companion animals and even aquatic animals. In recent years, it has been found that beyond gut health and immunoregulation, lactobacilli strains also play a role in the regulation of lipid metabolism.

Dysregulated lipid metabolism represents a critical risk for multiple production-related problems in livestock animals, such as excessive fat deposition, fatty liver syndrome and reduced feed utilization efficiency [5]; meanwhile, lipid metabolic disturbance also contributes to obesity and related metabolic diseases in companion animals [6], as well as abnormal lipid nutrition-related growth performance in aquatic species [7]. These effects bring huge economic losses to the aquaculture and livestock industries and represent a huge threat to the health of pets.

Given the conserved lipid regulatory signaling pathways shared by these different animal taxa, it is scientifically valuable to summarize the current progress of lactobacilli–lipid metabolism research across pigs, poultry, companion animals and aquatic animals within one review framework, which could identify specific strain–host interaction and cross-species research gaps that cannot be easily observed in single-species summaries.

While many excellent reviews have focused on the intestinal or immune-related functions of lactobacilli [8,9,10], few articles have comparatively showed the molecular mechanisms by which different lactobacillus strains modulate lipid metabolism in different animals. Therefore, this review specifically centers on lipid metabolism as its core theme. Herein, we summarize the latest findings regarding lactobacillus-mediated regulation of lipid absorption, lipogenesis, lipolysis and fatty acid oxidation in pigs, poultry, companion animals and aquatic species. We further discuss the underlying probable mechanism based on the gut–microbiota–liver axis in different animals. Finally, the existing limitations, unresolved questions and promising future directions for lactobacillus-based probiotic strategies in animal production are highlighted.

## 2. Application of Lactobacilli in Monogastric and Aquatic Animals

In monogastric (e.g., pigs and poultry) and aquatic animals, studies have demonstrated that dietary supplementation with lactobacilli can significantly enhance growth performance [11,12,13], improve both immunity and gastrointestinal health [12,14,15,16], and improve meat and egg quality [3,17,18,19]. Major health concerns for many monogastric animals are obesity and fatty liver disease. In fact, fatty liver is a prevalent issue in monogastric animals, particularly when fed high-energy diets (e.g., high-fat-content diets). Research has shown that lactobacilli can reduce the accumulation of fat in the liver in several monogastric species. By optimizing lipid metabolism, lactobacilli enable animals to utilize dietary lipids more efficiently, converting them into energy and body components with greater efficacy.

### 2.1. Swine

In swine, numerous studies have focused on the effects of lactobacilli on gut health and immune function, particularly in weaned pigs under severe-diarrhea conditions. In growing pigs, meat quality was also researched, and it could be related to many factors, including muscle fiber characteristics, oxidative stability, genetics, nutrition, and postmortem metabolism. Furthermore, it was indicated that meat quality of swine was also related to lipid metabolism [20]. The effects of lactobacilli on lipid related metabolism in pigs are summarized in Table 1.

These studies have demonstrated that lactobacilli can enhance intestinal barrier function in weaned pigs [11]. Additionally, it has been reported that specific strains of lactobacilli can improve average daily gain and feed conversion ratio in pigs while reducing serum concentrations of total cholesterol (TC), total bile acids (TBAs), triglycerides (TG), and low-density-lipoprotein cholesterol (LDL-C) [20]. These effects may be attributed to improved lipid digestion, absorption, and/or metabolism.

### 2.2. Poultry

There as a been a particular focus on reducing fatty liver syndrome, as this disease is prevalent in poultry, including laying hens and broilers; it poses significant threats to health and can even cause death [36]. The physiological factors of FLS in layers and broilers are totally different, for in the former, it is closely associated with their reproductive physiological characteristics [37], while in the latter, it is linked with their growth-oriented physiological traits [38]. Long-term feeding high-energy diets exceeds the metabolic capacity of broilers, leading to abnormal lipid metabolism and severe liver fat deposition, while in layers, high-energy diets could probably lead to liver hemorrhage. Several studies have also evaluated the effects of supplementing poultry diets with lactobacilli (Table 2). But because the liver metabolism mechanism could be different in layers and broilers, the effects of lactobacilli could be different. Generally, lactobacilli play a role by affecting gut microbiota and its metabolism, like bile acids and related enzymes, probably further affecting liver metabolism. It was reported that estrogens in serum could be increased by using lactobacilli [39], which showed that lactic acid bacteria could affect ovarian function in layers.

Table 2 summarizes the effects of lactobacilli on lipid metabolism in broilers and laying hens. In broiler chickens, dietary inclusion of lactobacilli has been associated with a more favorable lipid profile.

Egg quality, specifically cholesterol concentrations in eggs, which are closely related to lipid metabolism, is a primary focus in laying hens. Studies have reported that lactobacilli supplementation can enhance egg quality (as shown in Table 2). Additionally, lactobacilli can regulate the levels of various lipid components in the body, including serum total TC, TG, LDL-C, and high-density-lipoprotein cholesterol (HDL-C) [40].

### 2.3. Pets

Obesity is a significant health concern for pets, especially dogs and cats. There are many influencing factors, including high nutrition intake, physiology, genetics, intestinal flora, and endocrine diseases [60], and it is particularly prevalent in older animals. Similar to humans, obesity in pets can lead to various complications, such as diabetes, arthrosis, respiratory problems, cardiovascular diseases, and digestive disorders [61]. Obesity is also closely related to lipid metabolism disorders.

The beneficial effects of lactobacilli have been well documented in dogs, particularly in addressing diarrhea [62] and other health issues, including oral health [63], prevention of atopic dermatitis [64], and immune system enhancement [65,66,67] Similarly, in cats, lactobacilli have demonstrated immunoregulation and gut health modulation effects [68,69]. However, research on the impact of *lactobacilli* on lipid metabolism in dogs and cats remains limited.

Several studies have shown that lactobacilli may have potential applications in managing obesity in pets by modulating lipid metabolism (as summarized in Table 3). In dogs, *lactobacillus* treatments were linked with decreases in bodyweight, subcutaneous fat mass, and circulating cholesterol concentrations [70,71,72]. In cats, limited studies showed that *Lactobacillus* spp. could decrease the TG contents in serum [73,74], but the parameters were very limited and only focused on blood, without considering subcutaneous fat content and fat digestibility. Also, the test period was only 28 days, so longer periods should be implemented in future. These findings suggest that *Lactobacillus* spp. could be a valuable tool in future strategies to address obesity in dogs and cats.

### 2.4. Aquaculture

Numerous studies have demonstrated the beneficial effects of *L.* spp. on immune function in aquaculture, particularly in species such as carp and shrimp [75,76,77,78,79]. However, research on lipid metabolism in these species remains limited. For example, *L. casei* has been shown to protect the liver in common carp fry from damage caused by iron oxide nanoparticles (IoNPs), with no abnormalities having been observed in neither normal nor probiotic-based treatments [80]. Additionally, postbiotics derived from lactobacilli have gained significant attention in recent years. These postbiotics have been shown to improve liver health and reduce liver TG levels in common carp (*Cyprinus carpio*) fed a high-fat diet [71], indicating that inactivated lactobacilli can exert beneficial effects through their metabolites or bacterial components.

Table 4 summarizes the current applications of lactobacilli in aquaculture, focusing on lipid metabolism. These findings suggest that lactobacilli could play a crucial role in future strategies for improving lipid metabolism and overall health in aquaculture species. But most studies focused on lipid metabolism, without considering meat quality in aquaculture.

Despite the beneficial effects of lactobacilli reported in most investigations, inconsistent outcomes remain across studies. Such different responses can be largely attributed to multiple critical variables, including probiotic strain specificity, applied dosage, basal dietary composition, host species, and experimental duration. Even closely related lactobacillus strains may exert distinct regulatory effects on SCFA production. In addition, differences in feed nutrient background, animal model, and the length of intervention further complicate the comparability of the available results.

## 3. Mechanism

**Figure 1:**
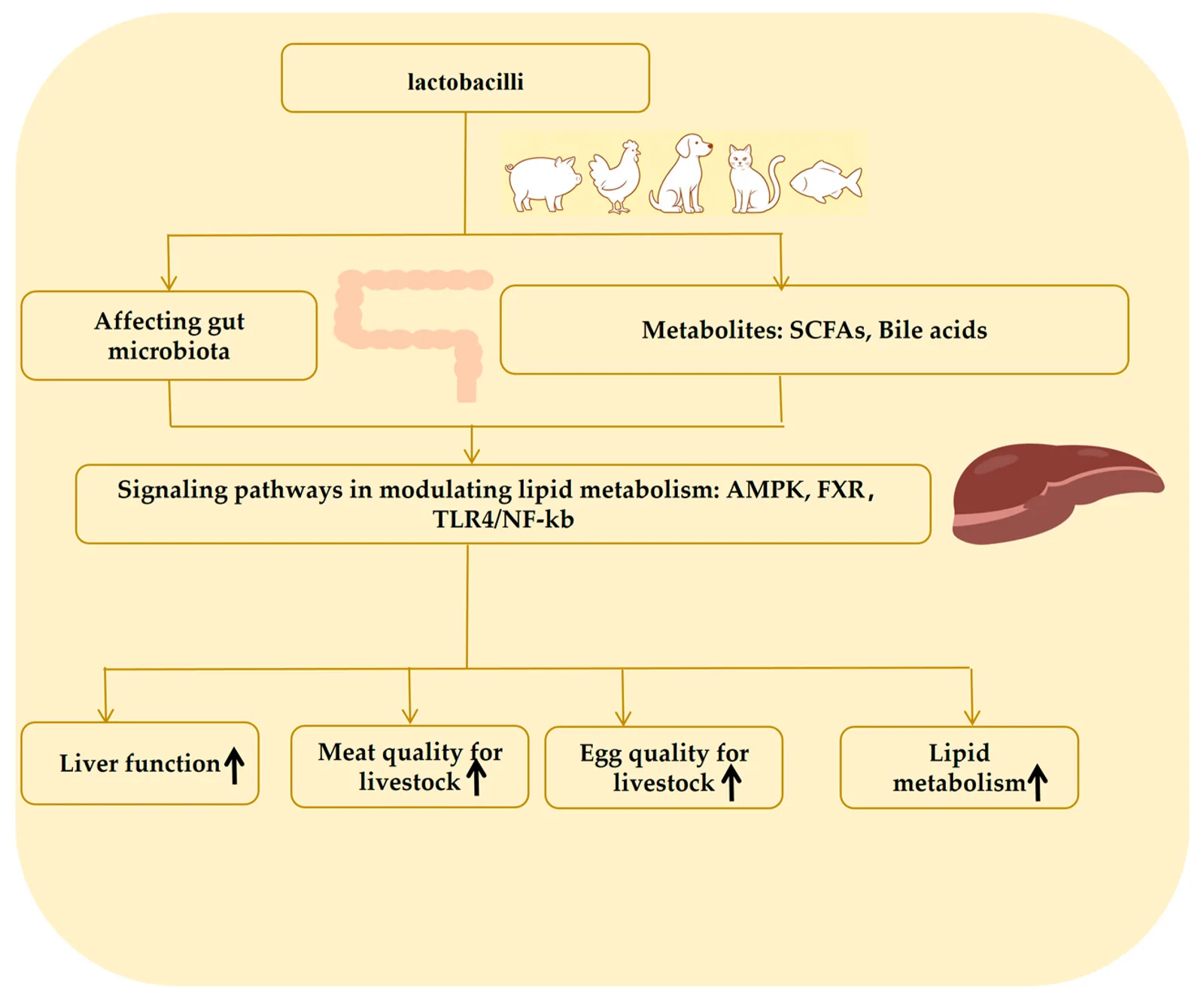
Lactobacilli play a role in the relationships of the gut–liver axis. Notes: **↑** meant up-regulation.

Although the modulatory mechanisms of *L*. spp. on lipid metabolism have been extensively elucidated in murine models, most frequently using high-fat-diet or hyperlipidemia-induced obese mice, these robust mechanistic findings cannot be directly and indiscriminately extrapolated to livestock, aquatic animals, or companion animals. Murine studies have clearly verified that lactobacilli regulate lipid metabolism via multiple conserved signaling pathways, including the AMPK, PPAR-α, Nrf2, and TLR4/NF-κB pathways (Figure 1) [87,88,89,90]. However, it should be emphasized that such pathway-centered mechanistic evidence is predominantly derived from rodent experiments. In contrast, the corresponding regulatory mechanisms in livestock, fish, and pets remain largely hypothetical. Direct cross-species extrapolation is currently lacking sufficient experimental validation due to interspecific differences in digestive physiology, gut microbiota, and lipid metabolic pathways. Furthermore, existing studies, whether in rodents or non-rodent animals, mainly focus on macroscopic pathway changes, while the key intermediate metabolites linking lactobacilli intervention and lipid metabolic regulation remain poorly clarified. Future research is therefore required to identify core metabolic intermediates and verify species-specific regulatory mechanisms, so as to improve the mechanistic rationality of cross-species application of *lactobacillus*-based lipid regulation.

### 3.1. Regulation of Signaling Pathways in Modulation of Lipid Metabolism

Meat quality in both broiler chickens and pigs could be influenced by dietary supplementation with *L.* spp. [3]. In broilers, the up-regulation of stearoyl-CoA desaturase-1 (SCD-1; Δ9-desaturase) enhanced the conversion of saturated fatty acids (SFAs) into monounsaturated fatty acids (MUFAs), thereby modifying the fatty acid composition of chicken meat through a shift from SFA dominance to MUFA enrichment. A probiotic was also indicated to modulate meat constitution by up-regulating ATGL, CPT-1, and HSL expression levels in the PMM in pigs [35].

AMPK pathway. As mentioned above, the AMPK pathway is wildly studied in lipid metabolism in mice and rats. In poultry, dietary supplementation with *L.* spp. has been found to up-regulate the AMPK-α/PPAR-α/CPT-1 pathway in the liver [44], which showed that *lactobacilli* could also modulate the AMPK pathway in broilers. Whether this remains true in other mammalian species remains to be investigated.

Farnesoid X receptor (FXR) pathway. This pathway is closely associated with bile acids and related metabolites, which play an important role in lipid metabolism. Certain *lactobacillus* strains have been shown to reduce the expression of lipid transporters in the intestine, thereby decreasing lipid uptake. *L. paracasei* led to the accumulation of cholesteryl-esters by antagonizing the LXR pathways in pigs def a high-fat diet [91]. Furthermore, *L. delbrueckii* was indicated to regulate the gut–liver FXR-FGF19 axis by down-regulating the expression of lipid metabolism-related genes, including ileal apical sodium-dependent bile acid transporter (ASBT), ileal bile acid-binding protein (IBABP), hepatic farnesoid X receptor (FXR), fibroblast growth factor (FGF19), and small heterodimer partner (SHP), but up-regulating hepatic high-density-lipoprotein receptor (HDLR), low-density-lipoprotein receptor (LDLR), sterol regulatory element-binding protein-2 (SREBP-2), and cholesterol-7alpha hydroxylase (CYP7A1) expression; this indicated that *L. delbrueckii* promoted ileal bile acid deconjugation with subsequent fecal total cholesterol and total bile acid extraction by modifying ileal microbiota composition and inducing hepatic bile acids synthesis in growing–finishing pigs [23].

Notably, current evidence reveals obvious limitations on universality and replicability. First, all above-mentioned valid FXR mechanistic studies are confined to individual species, and no replicated trials have verified whether such *lactobacillus*–FXR regulatory patterns are conserved in poultry or other species. Also, most existing studies focus only on phenotypic pathway changes at the mRNA levels, lacking verification of core intermediate metabolites and protein-level validation, which further weakens the robustness of current mechanistic evidence.

Other pathways. *L. salivarius* SNK-6 improved hepatic lipid mobilization and attenuated NAFLD-associated liver damage in laying hens, partly through modulation of the miR-130a-5p/MBOAT2 signaling pathway [92]. In broilers, *L. johnsonii* was found to modulate lipid metabolism by down-regulating key genes involved in lipogenesis, including lipoprotein lipase (LPL) in adipose tissue and stearoyl-CoA desaturase-1 (SCD-1), sterol regulatory element-binding protein-1c (SREBP-1c), and fatty acid synthase (FAS) in the liver [45]. Simultaneously, peroxisome proliferator-activated receptor alpha (PPARα), acyl-CoA oxidase (ACOX), and carnitine palmitoyltransferase-1 (CPT-1α) were up-regulated in hepatic tissue. These transcriptional changes were associated with elevated serum HDL cholesterol, consistent with enhanced lipid metabolism and reduced fat deposition [45].

### 3.2. Regulation of Intestinal Flora and SCFAs

Many of the positive effects of dietary lactobacillus treatments may be partly mediated by changes in the intestinal microbiome. Indeed, supplementation with lactobacilli can alter intestinal microbial composition, increasing the abundance of beneficial taxa and short-chain fatty acid (SCFA)-producing bacteria, which may contribute to improvements in lipid metabolism. They can also inhibit the growth of harmful bacteria and reshape the host intestinal microbial community through competitive exclusion, production of antimicrobial substances and regulation of intestinal pH.

The gut microbiota and its metabolites, like SCFAs, also play a pivotal role in host lipid metabolism. It has been implied that *lactobacilli* could modulate lipid metabolism in animals by altering intestinal microbiota composition and subsequently influencing SCFA metabolism. The primary SCFAs include acetate, propionate, butyrate, isobutyrate, valerate, and isovalerate [93,94]. Changes to the concentrations of these SCFAs have the potential to directly affect systemic lipid metabolism. Butyrate serves as an energy source for colonic epithelial cells [95], activates the AMPK/Sirt1/PGC-1α pathway to enhance mitochondrial function [96], and promotes systemic energy metabolism. Both acetate and propionate activate the AMPK pathway while suppressing fatty acid, cholesterol, and triglyceride (TG) synthesis [97]. These SCFAs also inhibit adipocyte activity through G protein-coupled receptor 43 (GPR43) activation, thereby reducing circulating fatty acid concentrations [98]. As the primary source of SCFAs, gut microbiota provide key enzymes for SCFA biosynthesis [99].

Lactobacillus-mediated modulation of gut microbiota in monogastric animals and aquatic species represents a major pathway for lipid metabolism regulation by altering SCFA levels. For instance, *L. casei* significantly increase total SCFAs, specifically propionate and valerate, in porcine intestines [100,101]. Artificial administration of sodium acetate, propionate, butyrate, or mixed SCFAs can reduce TG, total cholesterol (TC), and LDL-C levels in blood and liver, as well as increasing serum GLP-1, PYY, and leptin concentrations; this implies that SCFAs can modulate lipid metabolism and appetite-regulating hormones [102,103]. In broilers, volatile fatty acids (VFAs), such as acetate, propionate, and butyrate, play crucial roles in cecal microbiota development by suppressing Enterobacteriaceae and Salmonella growth [104]. Sodium butyrate (NaB) has also been shown to alleviate hepatic steatosis through AMPK/PPARα pathway activation, down-regulating lipid synthesis genes (SREBF1, ACC1, FASN, SCD1, LXRα, and HMGR) and promoting fatty acid oxidation [105].

Research suggests that probiotic lactobacilli can modulate host lipid metabolism through the production of short-chain fatty acids (SCFAs), particularly acetate and butyrate. In vitro cecal models showed that *L. salivarius* salicinius JCM 1230 and *L. agilis* JCM 1048 significantly increased propionate and butyrate production, which was accompanied by inhibition of pathogenic bacteria [106]. Combined use of *L. reuteri* PTA5-F13 and glycerol was found to enhance butyrate production in chicken cecal microbiota, which in turn improved lipid metabolism [107]. Treatment of immortalized chicken preadipocytes (ICP2) with butyrate (4 mM) reduced triglyceride and total cholesterol concentrations, suppressed expression of adipogenic genes (FABP4, C/EBPα, PPARγ, and LPL), and decreased lipid synthesis proteins [108]. In canines, another probiotic, *Bifidobacterium lactis* IDCC 4301, has been shown to alleviate diet-induced metabolic disorders by enhancing SCFA production, which, through acetate/butyrate-mediated dopamine stimulation, promotes glycolysis and ATP generation, and restoring glucose/insulin tolerance [109]. In crustaceans, lactobacilli and acetate emerged as potential intervention targets for improving lipid tolerance via bile acid and SCFA modulation [110]. Probiotic *L. rhamnosus* counteracted perfluorobutanesulfonate-induced bile acid dysregulation in zebrafish by enhancing fatty acid synthesis and β-oxidation [111]. Together, these studies suggest that probiotic lactobacilli can increase SCFAs and that SCFAs such as acetate and butyrate play an important role in regulating lipid metabolism through effects, including anti-inflammatory effects [112], across multiple levels, from microbial activity to adipocyte function.

However, the magnitude of SCFA increase following lactobacillus supplementation is not consistent across studies, even within the same species, and strain-, dose- and diet-dependent effects are evident. Therefore, generalizing SCFA-driven mechanisms across species should be done cautiously, and more standardized, multi-species replicated studies are needed to distinguish conserved responses from model-specific findings.

### 3.3. Intestinal Barrier and Lipid Metabolism in Current Models In Vivo and Vitro

Human cell sources are often used for mechanism studies for their stability. The HepG2 cell model was used to evaluate the modulation of *lactobacilli* in vitro. By establishing an NAFLD (non-alcoholic fatty liver disease) cell model, both *L. plantarum* AR113 and *L. casei* pWQH01 appeared to improve steatosis in a bile salt hydrolase (BSH)-dependent manner.

Primary chicken intestinal epithelial cells were isolated from 18-day-old chicken embryos and tested for lipid metabolism effects exerted by lactobacilli [44]; the results showed that TC and TG could be reduced in intestinal epithelial cells, but further pathways were not mentioned. Primary cells from monogastric animals are not stable, which limits the application of such cells.

In recent studies, more methods, including metabonomics, were used to study lipid metabolism in monogastric and aquatic animals. But due to limits such as cost and convenience, the mechanism was mostly studied in mice or rats. More studies about the lipid modulation mechanism need to be implemented in corresponding target animals.

## 4. Conclusions

Multiple lactobacillus strains exert beneficial regulatory effects on lipid metabolism across monogastric livestock, companion animals and aquatic species via multi-faceted pathways. Nevertheless, the overall strength of existing mechanistic evidence remains relatively weak, since most regulatory conclusions are extrapolated from rodent studies rather than being validated directly in target farmed animals. Several critical knowledge gaps still persist, including strain-specific functional differences, species-dependent responses, and the precise crosstalk between short-chain fatty acid signaling and the liver–adipose axis.

Above all, future work should focus on verifying species-specific *lactobacillus* regulatory mechanisms, exploring the main metabolic intermediates involved in bile acid–SCFA–gut microbiota signaling axes, and conducting multi-species replicated trials to improve the robustness of current mechanistic evidence.

## Figures and Tables

**Table 1:** The effects of lactobacilli on lipid metabolism in swine.

Animal Breed	Test Period	Lactobacilli ^#^ Species	Dose	Significant Serum/Plasma								
				TC ^a^	TG	LDL-c	HDL-c	TBAs	FFAs	Liver	Pork Quality	Ref.
Growing pigs												
Male pigs	175 d	*L. reuteri*	5.0 × 10^10^ CFU/kg feed	-	-	-	-	-	↑	-	↑	[19]
Cross-bred male pigs	15 d of hypercholesterolemia induction and 20 days of bacterium treatment	*L. acidophilus*, *L. casei*, and *Bifidobacterium longum*	3.0 × 10^7^ CFU/kg feed	↓	-	-	-	↓	-	↑ hypercholesterolemia improved	-	[21]
Female pigs	3 m	*L.* sp.	1% (*L. Buchneri* 3.0 × 10^7^ CFU/g; *L. plantarum* 1.6 × 10^8^ CFU/g; *L. casei* 1.4 × 10^8^ CFU/g)	↓	-	-	-	-	-	↑ Liver function	-	[22]
Barrows	28 d	*L. delbrueckii*	*0.1% diet* (1.4 × 10^8^ CFU/g)	↓	↓	-	-	↓ (serum) ↑ (fecal)	-	*FXR* ↓, *FGF19* ↓, *CYP7A1* ↑	-	[23]
Female Yorkshore Gilts	4 w	*L. acidophilus*	5 × 10^10^ CFU/g	↓	-	-	-	-	-	-	-	[24]
Yorkshire barrows	15 d (high-cholesterol diet) + 15 d (common diet)	*L. acidophilus*	2.5 × 10^11^ CFU per feed	-	↓	-	-	-	-	-	-	[25]
Cross-bred pigs (growing pigs)	42 d	*L. acidophilus*	1.0 × 10^9^ CFU/kg feed	-	-	↑	-	↑	-	-	-	[26]
Growing pigs	42 d	*L. plantarum* ZJ316	1 × 10^9^ CFU/kg feed	-	-	-	-	-	-	-	↑	[27]
Growing pigs	60 d	*L. reuteri*	2 × 10^9^ CFU/kg feed	-	-	-	-	-	-	-	↑ IMF	[28]
Growing pigs	12 w	*L. brevis*, *L. reuteri*, *L. paraplantarum*, and *L. pentosus*	1 × 10^9^ CFU/kg feed	-	-	-	-	-	-	-	↓ Cooking loss	[29]
Growing pigs	56 d	*L. amylovorus*	1 × 10^8^ CFU/kg feed	-	-	-	-	-	-	-	↑ Pork quality; ↑ PUFAs	[30]
Growing pigs	70 d	*L. jensenii*	1 × 10^9^ CFU/kg feed	-	-	-	-	-	-	-	↑ Meat juiciness and sensory palatability; ↓ Shear force	[31]
Weaned pigs												
Weaning pigs	6 w	*L. lactis*	0.01%, 0.02%, 0.03%	-	-	-	-	-	-	-	↑ GE Digestibility	[3]
Weaned piglets	21 d	*L. plantarum*	1.0 × 10^9^ CFU/kg feed	-	-	-	-	-	-	-	Δ tryp metab. Δ fat digest. & metab.	[32]
6d old weaned piglets	21 d	*L. frumenti*	1.0 × 10^8^ CFU/kg feed	-	-	-	-	-	-	-	Fatty acid β-oxidation-related metabolites in serum ↑	[33]
Hypercholesterolemic Landrace	6 w	*L. acidophilus* fructooligosaccharide, inulin, and mannitol	1 × 10^9^ CFU/d/pig	↓	↓	↓	-	-	-	-	-	[34]
Sows and offspring piglets	Sows: 85 d; Piglets: 21 d	*L. plantarum* *Saccharomyces cerevisiae*	1 × 10^9^ CFU/kg feed	-	-	-	-	-	-	-	i.m. fat ↓, i.m. PUFAs ↑	[35]

^a^ (Abbreviations in Table 1): ↑: up-regulation; ↓: down-regulation; “-”: no effects or not tested; TC: total cholesterol; TG: triglycerides; LDL-c: low-density-lipoprotein cholesterol; HDL-C: high-density-lipoprotein cholesterol; TBAs: total bile acids; FFAs: free fatty acids; PUFAs: polyunsaturated fatty acids; GE: gross energy; IMF: intramuscular fat; i.m.: intramuscular. ^#^, lactobacilli included the following species: *Lactiplantibacillus* (L.) *plantarum* (formerly *Lactobacillus plantarum*); *Limosilactobacillus* (L.) *reuteri* (formerly *Lactobacillus reuteri*); *Lacticaseibacillus* (L.) *rhamnosus* (formerly *Lactobacillus rhamnosus*); *Lacticaseibacillus* (L.) *brevis* (formerly *Lactobacillus brevis*); *Lactobacillus* (L.) *acidophilus*; *Lactobacillus* (L.) *delbruecki*; *Lantiplantibacillus* (L.) *pentosus* (formerly *Lactobacillus pentosus*); *Lactobacillus* (L.) *amylovorus*; *Lactobacillus* (L.) *jensenii*; *Limosilactobacillus* (L.) *fermentum* (formerly *Lactobacillus fermentum*); *Lactobacillus* (L.) *cripatus*; *Lactobacillus* (L.) *gasseri*; *Lactobacillus* (L.) *johnsonii*. The same rules apply in the following tables.

**Table 2:** The effects of lactobacilli on lipid metabolism in poultry.

Animal Breed	Test Period	Lactobacilli	Dose	Significant in Serum/Plasma						Liver	Other Relevant Effects	Ref.
				TC ^b^	TG	LDL-c	HDL-c	TBAs	FFAs			
Broilers												
1 d old chicks	35 d	*L. casei*	1.2 × 10^9^ CFU/mL	↓	-	↓	↑	-	-		-	[40]
1 d old chicks	6 w	*L. salivarius*	10^7^, 10^8^ and 10^9^ CFU/kg of feed	-	-	-	-	-	-	↑ Liver function	↑ Meat quality	[41]
7 d old SPF chicks	42 d	*L. rhamnosus*	2 × 10^8^ CFU/g	-	-	-	-	-	-	-	↑ Meat quality	[42]
1 d commercial broiler chickens	42 d	*L. acidophilus*	0.1% or 0.2% MOS + 10^6^ or 10^7^ LBA/g feed	-	-	-	-	-	-	-	HC/hypolipidemic effects were implied	[43]
Newly hatched	42 d	*L. johnsonii + L. crispatus*		↓	↓	-	-	-	-	-	↓ Abdominal fat deposition	[44]
1 d old chicks	42 d	*L. johnsonii*	1 × 10^8^ CFU/g feed	-	↓	↓	-	-	-	-	↓ Abdominal fat deposition	[45]
1 d old chicks	42 d	*L. salivarius*	1 × 10^9^ CFU/g	↓	↓	↓	-	-	-	-	↑ BW, ↑ BWG, and ↑ FCR	[46]
Newly hatched	42 d	*L. johnsonii*	1 × 10^8^ CFU/kg	-	-	-	-	-	-	Induced liver abnormality attenuated	-	[47]
	42 d	*L. plantarum*	1 × 10^8^ CFU/kg feed	-	-	-	-	-	-	↓ Lipid peroxidation	↓ Serum lactate dehydrogenase and AATs	[48]
1 d old chicks	42 d	*L.* spp. (12 strains)		↓	↓	↓	-	-	-	-	-	[49]
	21 d	*L. plantarum*	9 × 10^7^ CFU/embryo	-	↑	-	-	-	-	↑ TG, ↑ TC, ↑ HDL, and ↓ LDL	-	[50]
1 d old chicks	21 d	*L. rhamnosus*	1 × 10^9^ CFU/kg feed	-	-	-	-	-	-	↑ MMP13 and MMP gene expression	-	[51]
Chickens	35 d	*L. farciminis + L. rhamnosus*	4 g/10 kg of feed	↓	-	-	-	-	-	-	-	[52]
Hybrid	42 d	*L. fermentum*		-	↓	-	-	-	-	-	-	[53]
Layers												
	24 w	*L. acidophilus*		-	-	-	-	-	-	-	↓ TC in eggs	[54]
	12 w	Postbiotic metabolites from *L. plantarum* strains		↓	-	-	-	-	-	-	↓ TC in yolk; ↓ fecal SCFAs	[55]
Jing-hong No. 1	12 w	*L. rhamnosus*	8 × 10^10^ CFU/kg feed	-	↓	↓	-	-	↓	↓ TG	-	[56]
Hy-line hybrid	24 w	*L. acidophilus*										[57]
Hy-line brown	8 w	A high-GABA-producing *L. strain*		-	-	-	-	-	-	-	↓ TC in eggs	[58]
Hy-line brown	16 w	*L.* *acidophilus*		↓	↓	↓	↑	-	-	-	-	[59]
Hy-line brown	10 w	*L. plantarum*	1 × 10^9^ CFU/kg feed	-	-	-	-	-	-	↓ Hepatic lipid accumulation	-	[39]

^b^ (Abbreviations in the table): ↑: up-regulation; ↓: down-regulation; “-”: no effects or not tested; TC: total cholesterol; TG: triglycerides; LDL-c: low-density-lipoprotein cholesterol; HDL-C: high-density-lipoprotein cholesterol; TBAs: total bile acids; FFAs: free fatty acids; BW: body weight; BWG: body weight gain; FCR: feed conversion ratio; AATs: amino acid transporters.

**Table 3:** The effects of lactobacilli on lipid metabolism in pets.

Breeds	Test Period	Lactobacilli	Dose	Effects ^c^	References
Dogs	7 d	*L. fermentum* AD1	10^9^ CFU	↑ Total lipid (serum) ↓ Glucose (serum)	[70]
	10 w	*L. gasseri* BNR17 and *L. plantarum*	2 × 10^9^ CFU/g + over 1 × 10^9^ CFU/g	↓ Body weight ↓ BCS ↓ Subcutaneous fat mass and proportion at the level of the third lumbar vertebra	[71]
	28 d	*L. acidophilus*	2 × 10^8^ CFU/day	↓ TG (serum) No effects on body weight No effects on apparent nutrient digestibility	[72]
Cats	28 d	*L. plantarum* L11	10^9^ CFU/day	↓ TG (serum)	[73]
	28 d	*L. plantarum* L-27-2 and *Pediococcus lactis* L-14-1	10^9^ CFU/day + 10^9^ CFU/day for each bacterium	↓ TG, ↓ LDL-C ↑ HDL-C (serum)	[74]

^c^ (Abbreviations in the table): ↑: up-regulation; ↓: down-regulation; TG: triglycerides; LDL-c: low-density-lipoprotein cholesterol; HDL-C: high-density-lipoprotein cholesterol; BCS: body condition score.

**Table 4:** The effects of lactobacilli on lipid metabolism in aquatic animals.

Animals	Lactobacilli	Testing Period	Dose	Effects ^d^	References
Common carp	*L. casei*	-	10^6^ CFU/g; 10^7^ CFU/g	Liver in common carp fry damaged by iron oxide nanoparticles (IoNPs) was protected	[80]
Common carp (*Cyprinus carpio*)	*Cetobacterium somerae* and *L. lactis*	-	0.2, 0.3 g/kg feed	↓ TG ALT and AST (serum)	[81]
*Nile tilapia juveniles*	*L Plantarum* MR1	-	5% of body weight	High-carbohydrate diet-induced hepatic lipid accumulation was ameliorated	[82]
*Micropterus salmoides*	*L. casei* K17	8 w	1 × 10^8^ CFU/g feed	↓ TG (serum and liver) ↓ TC (serum and liver)	[83]
*Oreochromis niloticus*	*L. plantarum*	8 w	10^8^ CFU/g feed	↓ Liver damage	[84]
*Danio rerio*	*L. fermentum* E15	4 w	10^8^ CFU/g feed	↑ SCFAs ↓ Lipid deposition (liver and blood)	[85]
*Megalobrama amblycephala*	*L. plantarum*	8 w	2 × 10^8^ CFU/g feed	Bile acid recycle improved ↓ Liver lipid deposition	[86]

^d^ (Abbreviations in the table): ↑: up-regulation; ↓: down-regulation; TC: total cholesterol; TG: triglycerides; ALT: alanine aminotransferase; AST: aspartate aminotransferase; SCFAs: short-chain fatty acids.

## Data Availability

No new data were created or analyzed in the study. Data sharing is not applicable to this article.

## Abbreviations

SCFA	short-chain fatty acid
TC	total cholesterol
TBAs	total bile acids
TG	triglycerides
ALT	alanine aminotransferase
AST	aspartate aminotransferase
LDL-C	low-density-lipoprotein cholesterol
HDL-C	high-density-lipoprotein cholesterol
IoNPs	iron oxide nanoparticles
HFD	high-fat diet
SFA	saturated fatty acid
MUFA	monounsaturated fatty acid
AMPK	adenosine 5′-monophosphate (AMP)-activated protein kinase
ASBT	apical sodium-dependent bile acid transporter
IBABP	ileal bile acid-binding protein
FXR	farnesoid X receptor
FGF	fibroblast growth factor
SHP	small heterodimer partner
HDLR	high-density-lipoprotein receptor
LDLR	low-density-lipoprotein receptor
SREBP-2	sterol regulatory element-binding protein-2
CYP7A1	cholesterol-7alpha hydroxylase
LPL	lipoprotein lipase
SCD-1	stearoyl-CoA desaturase-1
SREBP-1c	sterol regulatory element-binding protein-1c
FAS	fatty acid synthase
PPARα	peroxisome proliferator-activated receptor alpha
ACOX	acyl-CoA oxidase
CPT-1α	carnitine palmitoyltransferase-1
VFAs	volatile fatty acids
NAFLD	non-alcoholic fatty liver disease
GLP-1	glucagon-like peptide-1
GPR43	G protein-coupled receptor 43
BSH	bile salt hydrolase
PMM	psoas major muscle
AFB1	aflatoxin B1
LDM	longissimus dorsi muscle
BCS	body condition score
